# Case of Neuroma

**Published:** 1863-01

**Authors:** Jehiel Stearns

**Affiliations:** Pompey, N. Y.


ART. V.—*Case of Neuroma.* *By* JEHIEL STEARNS, M. D., *Pompey, N. Y.*

I will take the liberty to state to you a case of *Neuroma*, which I think is of rare occurrence, as it is the only case that has come under my observation in an extensive practice of forty-five years. Mr. M. P., aged forty, was afflicted with neuralgic pains in the knee, leg and foot of the right limb. After two years' continuance he discovered a small tumor, about four inches above the ham, on the back side of the thigh, quite sensitive, and producing pain on handling. At this period I examined the case, found a hard, smooth, slightly oval tumor, about the size of a walnut, in the vicinity of the sciatic nerve. It admitted of motion laterally, but not in the direction of the nerve, and I could perceive no pulsation; therefore I

concluded that it was the nerve itself, or attached to it. It increased in size for about one year, and was very painful, when it became about as large as a hen's egg. At this time he consulted Dr. Mott of New York, who advised to have it removed. On his return I operated, making a free incision about six inches in length, exposing the tumor with a bright, smooth covering, on which I discovered a small nerve not divided, which I carefully dissected off and preserved, which I think helped very much to preserve the vitality of the limb. I then divided the main nerve above and below, and removed the tumor, which appeared to be a diseased growth of the nerve, but above and below it was in a normal state. The leg and foot was immediately paralyzed in a great degree; in a few days gangrenous blisters appeared on the foot, and with much difficulty the vitality of the limb was preserved; sensation and vitality gradually returned, and he is now able to walk and perform good business.

Dr. Mott requested me to send him the tumor, and he now has the specimen.

